# Perception of quality of health delivery and health insurance subscription in Ghana

**DOI:** 10.1186/s12913-016-1602-4

**Published:** 2016-07-29

**Authors:** Joshua Amo-Adjei, Prince Justin Anku, Hannah Fosuah Amo, Mavis Osei Effah

**Affiliations:** 1 African Population and Health Research Centre, Nairobi, Kenya; 2 Department of Population and Health, University of Cape Coast, Cape Coast, Ghana; 3 Department of Business Administration, Valley View University, Oyibi, Ghana; 4 Department of Accounting and Finance, University of Cape Coast, Cape Coast, Ghana

**Keywords:** Quality, Health care, Health insurance, Subscription, Ghana

## Abstract

**Background:**

National health insurance schemes (NHIS) in developing countries and perhaps in developed countries as well is a considered a pro-poor intervention by helping to bridge the financial burden of access to quality health care. Perceptions of quality of health service could have immense impacts on enrolment. This paper shows how perception of service quality under Ghana’s insurance programme contributes to health insurance subscription.

**Methods:**

The study used the 2014 Ghana Demographic and Health Survey (GDHS) dataset. Both descriptive proportions and binary logistic regression techniques were applied to generate results that informed the discussion.

**Results:**

Our results show that a high proportion of females (33 %) and males (35 %) felt that the quality of health provided to holders of the NHIS card was worse. As a result, approximately 30 % of females and 22%who perceived health care as worse by holding an insurance card did not own an insurance policy. While perceptions of differences in quality among females were significantly different (AOR = 0.453 [95 % CI = 0.375, 0.555], among males, the differences in perceptions of quality of health services under the NHIS were independent in the multivariable analysis. Beyond perceptions of quality, being resident in the Upper West region was an important predictor of health insurance ownership for both males and females.

**Conclusion:**

For such a social and pro-poor intervention, investing in quality of services to subscribers, especially women who experience enormous health risks in the reproductive period can offer important gains to sustaining the scheme as well as offering affordable health services.

## Background

Healthcare financing remain a major concern for many countries globally, especially middle and low-income countries [1]. In the light of rising burden of non-communicable diseases (NCDs) coupled with the already high prevalence of infectious diseases, the challenges to healthcare financing has become more pressing. In many middle and low-income settings, financing healthcare is worsened by the wide socioeconomic inequalities [2, 3]. Health insurance, which involves pooling risk and resources, is widely regarded as a mean to ensure equity in health care, protect households from catastrophic health spending and improve access to healthcare especially among the poor and vulnerable in society [4–6].

Health insurance in developing countries offers a great deal of opportunity for people, especially the poor to access healthcare anytime the need arises. However, in a number of countries where health insurance is largely regarded as a pro-poor intervention, enrolment is much lower than projections. Factors such as poverty, perceived poor quality of healthcare to clients on insurance, poor perception about the concept of health insurance, lack of information, high number of people in the informal sector, low levels of education, inefficient institutional coordination, lack of trust, among others have been documented as responsible for the low enrolment onto health insurance in many low-middle income countries [7–12].

Although Ghana’s national health insurance programme is considered one of the best performers in sub-Saharan Africa, a lot more need to be done to propel enrolment in order to reap the anticipated benefits for establishing the programme [13]. For instance, in 2011, while 31 % of females had never enrolled in the scheme, it was 44 % among males. Three key reasons for non-subscriptions among both sexes were; high premium (females: 39 %; males: 32 %), “no need for health insurance” (females: 21 %; 28 % for males) and “lack of trust in the NHIS” (females: 6 %; males: 5 %) [14]. The situation in 2011 had barely changed by 2014 – around 6 in 10 women and half of men were covered by health insurance [15]. Thus, sub-optimal enrolment is a major challenge towards achieving a nationwide coverage of the scheme.

In this paper, we discuss how perceptions of quality of healthcare delivery under the scheme impacts on subscription rates in Ghana, which is a detour from existing studies, that have looked at either perceptions of quality of health delivery under the scheme [16] or perceptions of benefits under Ghana’s health insurance [17]. While these studies advance our understanding of perceptions about the scheme, they failed to account for how these perceptions affect actual subscriptions. This study therefore becomes important in the light of frequent public criticisms about the performance of the scheme. For a scheme which relies extensively on membership subscriptions, negative perceptions about the quality of healthcare delivered to subscribers may negatively affect renewals and enrolments and ultimately, the survival of the scheme. To that extent, this study becomes useful for programmatic improvement and extending our understanding of the nuances involved in health insurance subscription in Ghana.

### Context of the study

At independence, the healthcare delivery system was modelled around the practice in Britain. Under this system, healthcare services provided in public institutions were virtually free to all citizens. Overtime, this practice changed due to decline in economic fortunes, which made free healthcare unsustainable. Fees for healthcare services were introduced in 1985 and subsequently, in 1992, the “cash-and-carry” system was introduced where patients had to make full payment for all services rendered at the various health facilities [18]. The “cash-and-carry” system soon became unpopular as it resulted in low utilization of healthcare services especially among the poor of the population. The deteriorating health situation in the country prompted government to look for alternative means of providing quality and affordable healthcare to all Ghanaians especially the poor and the vulnerable, leading the extension of the National Health Insurance Scheme (NHIS) nationwide in 2003 [19]. Prior to that there were some district level mutual health insurance schemes of which the Nkoranza model is popularly mentioned see, [20]. In essence, the policy was a pro-poor strategy of extending healthcare access to population hitherto would not be able to access health services easily arising from financing difficulties. The range of premiums charged currently ranges between GH₵7.2 and GH₵ 48 ≈ $1.9-$12.3, depending on whether one resided in a relatively developed urban area or not. Other means of subscribing are 2.5 % monthly Social Security and National Insurance Trust (SSNIT) remittance to the scheme on behalf of formal workers, explicit exemption from paying premiums by those categorized as vulnerable groups; the very poor, children below age 18, pregnant women, and people aged 70 years and above [21].

The scheme is run on the basis of a pre-defined package to subscribers and active members. Health conditions captured under the scheme cover about 95 % of the endemic diseases in the country. Despite the huge potential benefits that are associated with the NHIS, enrolment onto the scheme continues to be far from optimum with lower enrolment among the poor even though the scheme is designed as pro-poor [17, 22, 23]. On the account of this, studies aiming at understanding some specific “threats” to increased subscriptions are worth pursuing.

## Methods

This paper employed the 2014 Ghana Demographic and Health Survey (GDHS14) dataset to analyse our study variables. The GDHS14 was the sixth round of the surveys, which started in 1987/88 in a number of developing countries in Asia and Africa. The Ghana Statistical Service (GSS), the lead organisation responsible for this survey collaborated with the ICF Macro and the Ghana Health Service (GHS). The survey relied on multi-staged sampling, which involved the selection of 427 clusters/enumeration areas (EAs) drawn from the 2010 Population and Housing Census. Of the total EAs, 216 were drawn from urban areas across the 10 administrative regions of the country using random techniques. The same approach was used to draw 211 clusters/EAs from rural areas bearing regional proportions in mind. In these clusters, a total of 12,831 households were selected with equal number of households per cluster. As a result, the sample was not self-weighting at the national level but weighting factors were computed for each cluster/EA to help generate results that reflect the regional share of the national population. Inclusion criteria were being a male or female between ages 15–49 and 15–59 respectively; de factor resident in the selected and informed consent duly granted. At a response rate of 97 %, 9,656 females and 4,609 males (response rate 95 %) participated in the survey. The dataset is freely available for public use on www.measuredhs.com. The questionnaires used for the 2014 GDHS is also appended to the final report published by Ghana Statistical Service (GSS), Ghana Health Service (GHS) (15).

Each respondent provided informed verbal and written consent to participate in the study. For respondent below 18 years (the statutory age for defining adulthood), parental or legal guardian consent followed by respondent assent was secured. Ethical approval for the data collection was provided by the Ethical Review Committee of Ghana Health Service, Accra, Ghana and the Institutional Review Board of ICF International, USA.

### Study variables

The two key variables in this study are perception of service quality by holding an NHIS card (independent) coded as better = 1, same = 2, worse = 3 and “don’t know” =8 and ownership of an NHIS card (dependent variable) captured as “no” = 0 and “yes” = 1. However, for the sake of specificity, “don’t know” in the case of the first variable was recoded to missing. To help us clearly decipher how robust our key explanatory variable relates to ownership of health insurance package, some theoretically relevant variables are included in the analysis. These are education (no education = 0, primary = 1, secondary = 3 and higher = 4), ethnicity (Akan = 1, Ga/Dangme = 2, Ewe = 3, Mole-Dagbani = 4, Gurma = 5 and Others = 6), wealth (poorest = 1, poorer = 2, middle/average = 3 richer = 4, richest = 5), religion (Orthodox Christian = 1 [Catholic, Presbyterian, Methodist and Anglican], Pentecostal/Charismatic = 2, Other Christian = 3, Islam = 4 and Traditional/Spiritualist = 5). Other variables were region of residence (Western = 1, Central = 2, Greater Accra = 3, Volta = 4, Eastern = 5, Ashanti = 6, Brong-Ahafo = 7, Northern = 8, Upper East = 9 and Upper West = 10), type of place of residence (urban = 1, rural = 2), age (15-19 = 1, 20-24 = 2, 25-29 = 3, 30-34 = 4, 35-39 = 5, 40-44 = 6, 45-49 = 7, 50-54 = 8, and 55-59 = 9) and occupation (not working = 0, professional/managerial/clerical = 1, sales/services = 2, agricultural employee/employed =3 and skilled/unskilled manual = 4).

### Analytical procedure

The analysis proceeded on the assumption that if perception of service quality by holding an NHIS care was better, not different or worse, the effects will be observed on whether an individual had own health insurance or not. To verify this, we first explored the bivariate association between socioeconomic characteristics and perceptions of service quality by holding insurance card using descriptive statistics with corresponding Chi-square test of association. This followed by running a bivariate logistic regression between perception of quality and ownership of health insurance. The next level was to control for the effects of other possible factors on insurance subscription. Separate analyses were done for females and males, as the GDHS data files do not easily allow merging female and male datasets. The results are presented in odds ratios with values less than 1 indicating less likelihood of a respondent being subscriber while those above 1 indicate higher likelihood.

## Results

Of the 7,304 females who responded to the question on quality of service by holding NHIS card, about 33 % each perceived service to be better, same and worse. Just about the same pattern noted among females was observed among males; approximately 34 % thought that service under the scheme was better and worse respectively while 31 % of them perceived no differences in service quality between NHIS subscribers and non-card subscribers. Among males, a sligth majority (51 %) compared to an absolute majority of females (62.4 %) who were covered by the NHIS. Regarding other characteristics of sampled population, a higher proportion of females were in the richest wealth quintile (23 %), had attained secondary education (56.8 %), resident in the Greater Accra region (20.2 %), currently living with a man or married (56.6 %), living in an urban area (53.8 %) and were employed in the sales/services sector (38.8 %). The characteristics of males also followed similar trajectory except that in terms of occupation, agricultural workers constituted the highest proportion (31.8 %).

### Service quality by holding NHIS and socioeconomic variables

Tables 1 and 2 show bivariate association between socioeconomic variables and perceptions about quality of service quality by holding an NHIS card, disaggregated by sex. In terms of education and perception of quality, we observed that with rising education, females were more likely to rate service under the NHIS as worse. Perceptions about service quality by holding NHIS shifted towards worse with rising household affluence. Females resident in the Western region were more likely to perceive service quality by holding NHIS as better. However, females in the Ashanti region were more inclined to indicate that the service quality under NHIS as worse. While females resident in urban areas rated service quality to be worse, those in rural areas indicated it was better. Significant differences in perceptions about service quality among females also existed by age, religion, ethnicity, marital status and occupation (Table 1).

**Table 1:** Perception of service quality by holding NHIS by socioeconomic status among Ghanaian females

Explanatory variables	Perception of service quality by holding NHIS			
	Better (%)	Same (%)	Worse (%)	N
Highest educational level (*χ* ^2^ = 128.66; *p* < 0.000)				
No education	39.5	36.7	23.8	1,332
Primary	35.3	35.4	29.3	1,219
Secondary	33.1	31.1	35.8	4,265
Higher	19.5	35.7	44.8	487
Wealth index (*χ* ^2^ = 259.17; *p* < 0.000)				
Poorest	45.5	33.8	20.7	1,155
Poorer	42.2	33.3	24.5	1,214
Middle	35.5	31.9	32.6	1,496
Richer	29.4	33.1	37.5	1,670
Richest	22.9	33.7	43.4	1,767
Region (*χ* ^2^ = 722.82; *p* < 0.000)				
Western	56.0	31.7	12.3	841
Central	38.4	40.7	20.9	636
Greater Accra	18.4	42.6	39.1	1,237
Volta	28.6	31.4	40.0	598
Eastern	48.6	25.0	26.4	713
Ashanti	18.8	27.3	53.9	1,478
Brong-Ahafo	34.1	27.2	38.7	683
Northern	44.4	37.4	18.2	602
Upper East	41.7	41.9	16.3	326
Upper West	47.7	27.7	24.5	185
Type of place of residence (*χ* ^2^ = 154.54; *p* < 0.000)				
Urban	26.6	34.0	39.5	3,940
Rural	42.2	32.2	25.7	3,363
Current marital status (*χ* ^2^ = 12.45; *p* < 0.014)				
Never in union	34.8	31.9	33.4	2,279
Currently in union/living with a mans	33.6	33.8	32.5	4,314
Formerly in union/living with a man	30.9	33.3	35.8	710
Age (*χ* ^2^ = 36.35; *p* < 0.000)				
15–19	38.3	34.9	26.8	1,189
20–24	34.2	30.9	34.8	1,232
25–29	32.3	33.6	34.2	1,298
30–34	29.9	34.8	35.3	1,081
35–39	34.3	33.0	32.7	1,064
40–44	31.7	32.4	36.0	799
45–49	35.4	31.8	32.7	638
Religion (*χ* ^2^ = 80.88; *p* < 0.000)				
Orthodox	34.6	32.4	33.0	1,866
Pentecostal	30.3	33.5	36.3	2,920
Other Christian	36.4	28.6	34.9	1,054
Islam	37.6	35.4	27.0	1,181
Traditional/others	38.0	42.3	19.7	281
Ethnicity (*χ* ^2^ = 183.00; *p* < 0.000)				
Akan	33.8	31.0	35.2	3,665
Ga/Dangme	32.2	34.8	33.0	495
Ewe	28.0	33.3	38.7	938
Mole-Dagbani	41.6	32.1	26.2	1,160
Gurma	30.1	50.0	19.9	378
Others	31.0	35.7	33.2	620
Occupation (*χ* ^2^ = 117.76; *p* < 0.000)				
Not working	38.8	34.6	26.7	1,705
Professional/Managerial	23.9	30.9	45.3	533
Sales/Services	29.7	32.7	37.6	2,830
Agricultural worker	41.6	33.0	25.5	1,325
Skilled/Unskilled	31.5	33.4	35.1	896

Computed from 2014 GDHS

**Table 2:** Perception of service quality by holding NHIS by socioeconomic status among Ghanaian males

Explanatory variables	Perception of service quality by holding NHIS			
	Better (%)	Same (%)	Worse (%)	N
Highest educational level (*χ* ^2^ = 67.97; *p* < 0.000)				
No education	47.3	21.0	31.7	266
Primary	43.1	37.5	19.5	282
Secondary	32.0	31.3	36.7	1,692
Higher	29.3	32.3	38.4	383
Wealth index (*χ* ^2^ = 97.97; *p* < 0.000)				
Poorest	48.1	25.7	26.2	460
Poorer	41.4	30.7	27.9	442
Middle	34.9	29.9	35.2	478
Richer	26.0	34.7	39.3	573
Richest	27.0	32.7	40.3	670
Region (*χ* ^2^ = 420.75; *p* < 0.000)				
Western	19.6	30.5	49.9	292
Central	67.0	15.4	17.6	193
Greater Accra	17.5	25.8	56.7	401
Volta	35.6	32.2	32.2	209
Eastern	33.3	37.6	29.1	269
Ashanti	34.3	41.4	24.3	544
Brong-Ahafo	16.2	43.0	40.8	271
Northern	53.0	13.7	33.3	244
Upper East	63.8	28.4	7.8	123
Upper West	54.5	18.8	26.6	75
Type of place of residence (*χ* ^2^ = 29.80; *p* < 0.000)				
Urban	28.2	34.9	36.9	1,354
Rural	40.9	27.0	32.1	1,271
Current marital status (*χ* ^2^ = 10.75; *p* < 0.029)				
Never in union	35.1	33.3	31.6	1,118
Currently in union/living with a mans	34.4	29.0	36.6	1,387
Formerly in union/living with a man	27.2	34.0	38.7	120
Age (*χ* ^2^ = 38.01; *p* < 0.002)				
15–19	37.4	36.3	26.3	562
20–24	35.0	28.3	36.7	331
25–29	39.5	28.6	31.9	301
30–34	27.9	29.9	42.2	298
35–39	30.9	27.6	41.4	309
40–44	33.7	26.2	40.1	277
45–49	32.0	30.6	37.4	220
50–54	37.4	34.1	28.5	184
55–59	31.3	38.0	30.7	150
Religion (*χ* ^2^ = 32.52; *p* < 0.000)				
Orthodox	31.9	32.3	35.9	716
Pentecostal	34.5	31.2	34.3	742
Other Christian	26.5	34.3	39.1	458
Islam	41.0	27.3	31.7	507
Traditional/Others	43.7	28.5	27.8	200
Ethnicity (*χ* ^2^ = 132.48; *p* < 0.000)				
Akan	30.6	32.5	36.9	1,287
Ga/Dangme	26.0	33.0	41.0	179
Ewe	31.1	29.5	39.5	339
Mole-Dagbani	43.8	27.9	28.3	444
Gurma	55.0	23.5	21.5	158
Others	34.1	35.9	30.0	216
Occupation (*χ* ^2^ = 67.97; *p* < 0.000)				
Not working	35.1	37.0	27.9	408
Professional/Managerial	32.5	27.3	40.2	435
Sales/Services	29.7	32.7	37.6	278
Agricultural worker	41.4	27.3	31.3	819
Skilled/Unskilled	29.1	33.4	37.4	671

Computed from 2014 GDHS

Table 2 presents perceptions about service quality among males. While a higher proportion of males with no education (47 %) said service quality was better by holding NHIS, about 38 % of those with higher education thought service was worse with NHIS. The poorest among males were more likely to state that service quality by holding NHIS was better while richest males tended to rate NHIS services poor. Males in the Central region tended to rate service under the NHIS as better while their counterparts in the Greater Accra region classified service quality under the NHIS as worse. About two-thirds of males in professional/clerical/managerial employments indicated described NHIS services as worse.

### Multivariable analysis

In this section, we report on the impacts of perceptions of service quality by holding NHIS and insurance ownership. For females (Table 3) who rated NHIS service poorly were significantly less likely to have an insurance cover (OR = 0.453; 95 % CI = 0.375, 0.554). Similarly, males with similar views about the scheme were significantly less likely to have an insurance cover (OR = 0.697; 95 % CI = 0.515, 0.944) than those who rated the quality of health service as better using the NHIS payment option. After controlling other covariates of insurance ownership, the effects of negative perceptions on service quality under the NHIS remained significantly robust for females (OR = 0.456; 95 % CI = 0.373, 0.549) but not so for males (Table 3).

**Table 3:** Logistic regression results on perceptions of healthcare quality under NHIS and ownership of health insurance

	Females				Males			
Explanatory factors	Model I	95 % CI	Model II	95 % CI	Model III	95 % CI	Model IV	95 % CI
*Perception of NHIS quality*								
Better	1	[1, 1]	1	[1,1]	1	[1,1]	1	[1,1]
Same	0.708^**^	[0.574,0.874]	0.699^**^	[0.561,0.871]	0.868	[0.644,1.168]	0.973	[0.724,1.308]
Worse	0.453^***^	[0.373,0.549]	0.456^***^	[0.375,0.554]	0.697^*^	[0.515,0.944]	0.781	[0.575,1.062]
*Level of education*								
None			1	[1,1]			1	[1,1]
Primary			1.117	[0.902,1.384]			0.715	[0.476,1.073]
Secondary			1.325^**^	[1.087,1.615]			0.946	[0.664,1.347]
Higher/Tertiary			2.196^***^	[1.450,3.327]			1.456	[0.795,2.668]
*Wealth quintile*								
Poorest			1	[1,1]			1	[1,1]
Poorer			1.097	[0.831,1.446]			1.506^*^	[1.002,2.262]
Middle			1.181	[0.884,1.578]			1.465	[0.933,2.298]
Richer			1.201	[0.865,1.668]			2.159^**^	[1.289,3.614]
Richest			1.709^**^	[1.183,2.470]			3.047^***^	[1.661,5.590]
*Region*								
Western			1	[1,1]			1	[1,1]
Central			0.763	[0.521,1.116]			1.871	[0.968,3.617]
Greater Accra			1.272	[0.800,2.023]			2.051	[1.000,4.208]
Volta			1.268	[0.804,1.998]			3.314^**^	[1.601,6.863]
Eastern			1.260	[0.824,1.927]			1.317	[0.713,2.435]
Ashanti			0.470^***^	[0.317,0.696]			1.168	[0.651,2.097]
Brong-Ahafo			1.681^*^	[1.128,2.506]			1.849^*^	[1.094,3.126]
Northern			2.749^***^	[1.678,4.502]			2.498^*^	[1.222,5.105]
Upper East			0.973	[0.633,1.496]			7.344^***^	[3.485,15.48]
Upper West			9.494^***^	[3.753,24.02]			11.11^***^	[4.275,28.87]
*Type of residence*								
Urban			1	[1,1]			1	[1,1]
Rural			0.911	[0.718,1.156]			1.106	[0.763,1.603]
*Marital status*								
Never in union			1	[1,1]			1	[1,1]
Currently in union/living with a mans			1.373^**^	[1.125,1.676]			1.389	[0.938,2.057]
Formerly in union/living with a man			0.971	[0.739,1.276]			1.001	[0.565,1.774]
*Age cohort*								
15–19			1	[1,1]			1	[1,1]
20–24			0.760^*^	[0.608,0.950]			0.708	[0.493,1.016]
25–29			0.968	[0.735,1.274]			0.570^*^	[0.362,0.898]
30–34			1.088	[0.815,1.453]			0.578	[0.326,1.022]
35–39			0.990	[0.730,1.342]			1.127	[0.623,2.037]
40–44			0.922	[0.670,1.268]			0.774	[0.434,1.379]
45–49			1.341	[0.941,1.913]			1.141	[0.593,2.196]
50–54			–	–			1.033	[0.547,1.950]
55–59			–	–			0.873	[0.424,1.801]
*Religion*								
Orthodox Christian			1	[1,1]			1	[1,1]
Pentecostal			0.888	[0.751,1.051]			1.240	[0.921,1.669]
Other Christian			1.007	[0.787,1.288]			0.634^**^	[0.468,0.860]
Moslem			0.920	[0.706,1.200]			0.952	[0.638,1.419]
Traditional/Others			1.055	[0.716,1.554]			0.563^**^	[0.399,0.794]
*Ethnicity*								
Akan			1	[1,1]			1	[1,1]
Ga/Dangme			1.212	[0.825,1.781]			1.856^*^	[1.043,3.302]
Ewe			1.364	[0.972,1.912]			0.893	[0.533,1.498]
Mole-Dagbani			1.207	[0.900,1.619]			1.465	[0.912,2.354]
Gurma			1.589	[0.996,2.535]			0.581^*^	[0.339,0.997]
Others			1.332^*^	[1.001,1.773]			0.855	[0.513,1.425]
*Occupation*								
Not working			1	[1,1]			1	[1,1]
Professional/Managerial			1.124	[0.800,1.577]			0.813	[0.459,1.442]
Sales/Services			0.996	[0.826,1.201]			0.477^**^	[0.291,0.783]
Agricultural worker			0.788	[0.619,1.004]			0.429^***^	[0.272,0.676]
Skilled/Unskilled			0.858	[0.673,1.095]			0.452^***^	[0.289,0.706]
Currently pregnant								
No/unsure								
Yes			2.593^***^	[1.942,3.462]				
_Cons	5.905^***^	[5.001,6.973]	2.717^***^	[1.667,4.429]	4.928^***^	[3.867,6.280]	1.604	[0.727,3.539]
*AIC*	7410.6		6940.2		2713.8		2473.3	
*N*	7552		7551		2774		2774	

^*^ *p* < 0.05, ^**^ *p* < 0.01, ^***^ *p* < 0.00

On other covariates, whereas females with higher education (OR = 2.196; 95 % CI = 1.450, 3.327) were significantly more likely to own health insurance, males in the same category were not significantly different than their counterparts with other levels of education. With respect to wealth, males in the highest quintile (OR = 3.047; 95 % CI = 1.661, 5.590) significantly tended to own health insurance than females (OR = 1.709; 95 % CI = 1.183, 2.470) in similar category. The most outstanding result is the high odds of NHIS ownership in the Upper West region for both females (OR = 9.494, 95 % CI = 3.753, 24.02) and males (OR = 11.11; 95 % CI = 4.275, 28.87). Another finding worth highlighting is the less likelihood of males in sales/services (OR = 0.477; 95 % CI = 0.291, 0.783), agricultural sector (OR = 0.429; 95 % CI = 0.272, 0.676) and skilled/unskilled manual labour (OR = 0.452; 95 % CI = 0.289, 0.706).

## Discussion

This paper examined how health insurance subscription in Ghana is influenced/shaped by perceptions of health service quality when healthcare cost is paid for using NHIS card. This was necessary in the light of the overriding goal of a national health insurance programme in Ghana—creating wealth through health by making access equitable in the country. The results show that perception of service quality was strongly related to ownership of health insurance and this was more pronounced in females than males.

Overall, the results show similarity to previous research on reasons for non-enrolment in health insurance schemes, particularly in sub-Sahara Africa. Quality of services provided to subscribers compared to those making out-of-pocket payments remains significant. For instance, the key reason for non-subscription and declining membership in a mutual health organisation (MHO) in Guinea Conakry was the poor quality of care offered to members of the scheme at health facilities [24]. Related findings have been reported about a community health insurance (CHI) in Uganda [25].

In terms of total enrolment in the insurance programme, our results evidence higher prevalence of subscription among women than men. Reasons that may account for this include: first, women are somewhat risk averse than men. Among men, low perception of risk could be attributed to male ‘misconception’ of physical superiority and therefore are unlikely to participate in programmes, which seek to pool and share risks associated with cost of health services. Literature on adoption of innovation also point out that men generally are late adopters (e.g. [26]) and are unlikely to own an insurance cover.

Also, the high enrolment of women more than men may be an artefact of prevailing policy of free health care for pregnant women. Under Ghana’s health insurance policy, pregnant women are part of the exempt category. This is more plausible given the age inclusion criteria for males (15–59 years) and females (15–49y years). The majority of these women were either pregnant or had been in the last five years to survey. This position is further strengthened other results from the study – pregnant women at the time of the survey reported higher tendency of being a subscriber than those unsure or did not know they were pregnant; this is consistent with a similar study in Burkina Faso [27].

Regarding perception of service quality and NHIS subscription, our findings reveal the importance of improving quality of healthcare in general and particularly among subscribers given the pro-poor nature of the programme. Perhaps, the concurrent running of the scheme with out-of-pocket payment could be contributing to perception of poor quality to subscribers. Martin and McMillan [28] noted that running a health insurance programme contemporarily with out-of-pocket payments might result in strict implementation of prescription, which can expose subscribers of health insurance to clinical consequences and thereby affect views of quality. It is imperative that quality of care in terms of convenience, scheme administrative arrangements, and provider attitudes can enhance enrolment if clients are satisfied with services provided by insurance providers [29].

Psychological theory also offers plausible reasons for female-male differential judgement of quality and how this affects outcomes. For instance, Darley and Smith [30] note that while females process information more comprehensively, consider both subjective and objective attributes of services and observant of delicate cues, men, on the other hand, are selective in information processing, use heuristics more frequently and are likely to miss details. This may account for the gender differences. Atinga, Abiiro [31] for instance observed that women were more likely to drop out of the NHIS due to quality concerns than males in informal settlements in Accra, Ghana. In a context where women in the reproductive ages (sample for this study) seem to have more life threatening health needs challenges as a result of childbirth see, [32], the health insurance managers ought to be concerned about total quality management. In a previous study, [33] the authors found that women enrolled in the NHIS were more likely to utilize antenatal services than those not enrolled regardless of socioeconomic status.

One striking finding is the high prevalence of NHIS ownership in the Upper West region among both males and females, albeit it is the poorest region in Ghana [34]. This is a positive observation in the sense that the NHIS is a pro-poor intervention. The equity goal in respect of access to health care is enhance with more registration in high poverty incidence areas. Unfortunately, however, in another high poverty incidence region, the Upper East, females were unlikely to own a health insurance, although more vulnerable to health problems during the reproductive period. Another finding worth our comment is the lower proportion of women in Ashanti region being subscribers to the NHIS. Perhaps, the trial of capitation provider-payment system started in the region, which received a lot of public criticisms, might be responsible for the low coverage of females in the scheme. Once again, these findings highlight the distortions and inconsistencies in equity for a pro-poor policy such as the NHIS in Ghana. Indeed, removing costs which leads to inequities in access to healthcare is one of the cardinal objectives of the intervention. Consequently, more needs to be done to propel enrolment in high poverty index regions.

The findings also revealed regional-level heterogeneity in perception of quality of health services provided under the insurance. Quality of health services coverage in the country is characterised by significant inequities in favour of the southern portion of the country [35]. Despite this, respondents, women and men in the three northern regions (Northern, Upper East and West) generally had better perceptions of the quality of healthcare with NHIS than those in the other regions. Apparently, this may be due to differential access and experiences with higher standards of care, largely in the private sector compared to the public [36], which are less likely to be cited in any of the three northern regions than the other parts of the country [37]. Thus, those at areas with relatively larger pool of options may demand higher quality of services as compared to regions with limited alternatives.

Our findings show that among women, formal education offered better prospects for owning a health insurance but not really the case for males. Unlike a previous study [23] in Ghana where female education was less effective in predicting health insurance subscription, in this paper, the association observed widens significantly with increasing education. The effect of wealth in this study on women’s health insurance subscription was minimal. Thus, education appears more empowering to women than wealth. Kumi-Kyereme and Amo-Adjei (23) made similar observations regarding the minimal role of wealth in women’s health insurance subscription. On the other hand, among men, it is rather wealth, which substantially increased the odds of insurance ownership rather than education. Thus, we notice varying associations of education and wealth on health insurance subscription among men and women.

We also note that perception of quality declined with increased in wealth. In areas where there is high proliferation of private insurance options, the rich are inclined to subscribe to private than national insurance programmes [38] but that is not the case in Ghana. The more plausible reason that could be accounting for the wealth gradient in perception of quality is that they may have higher expectations of quality services that is underpinned by ability to pay, likelihood of being urban areas, better educated and exposure to varied standards of healthcare. Possibly too, with the scheme mainly funded through 2.5 % VAT, the rich may expect/demand more quality attributes since they contribute more to financing the scheme through taxes.

Despite the important findings made in this study, the limitations imposed by the data need to mentioning. First, by relying on a cross sectional data, it is impossible to account for unobserved heterogeneity. The key explanatory variable (perception of quality of health care using NHIS as payment method) for this paper was also measured as perception, which makes it subject. These perceptions maybe fueled by third-party narratives about previous services received and that calls for caution in interpreting the findings.

## Conclusion

This study has demonstrated some significant associations between perceptions of quality of health services to subscribers and health insurance subscription, especially among women. For such a social and pro-poor intervention, it is imperative for the management of the scheme to devote attention to the whole quality mix of services offered to subscribers, whether real or perceived given that whichever way (good or bad) subscribers look at the services rendered when paying for services with the NHIS can have either drive down or up member subscription.

## Abbreviations

DMHIS, District Mutual Health Insurance Scheme; GDHS, Ghana Demographic and Health Survey; NHIS, National Health Insurance Scheme; SSNIT, Social Security and National Insurance Trust.
